# Calreticulin: Endoplasmic reticulum Ca^2+^ gatekeeper

**DOI:** 10.1111/jcmm.17839

**Published:** 2023-07-09

**Authors:** Marek Michalak

**Affiliations:** ^1^ Department of Biochemistry University of Alberta Edmonton Alberta Canada

## Abstract

Endoplasmic reticulum (ER) luminal Ca^2+^ is vital for the function of the ER and regulates many cellular processes. Calreticulin is a highly conserved, ER‐resident Ca^2+^ binding protein and lectin‐like chaperone. Over four decades of studying calreticulin demonstrate that this protein plays a crucial role in maintaining Ca^2+^ supply under different physiological conditions, in managing access to Ca^2+^ and how Ca^2+^ is used depending on the environmental events and in making sure that Ca^2+^ is not misused. Calreticulin plays a role of ER luminal Ca^2+^ sensor to manage Ca^2+^‐dependent ER luminal events including maintaining interaction with its partners, Ca^2+^ handling molecules, substrates and stress sensors. The protein is strategically positioned in the lumen of the ER from where the protein manages access to and distribution of Ca^2+^ for many cellular Ca^2+^‐signalling events. The importance of calreticulin Ca^2+^ pool extends beyond the ER and includes influence of cellular processes involved in many aspects of cellular pathophysiology. Abnormal handling of the ER Ca^2+^ contributes to many pathologies from heart failure to neurodegeneration and metabolic diseases.

When all the parts of the puzzle start to look like they fit it. Van Morrison

## 1 INTRODUCTION

My encounter with calreticulin started in the 70s after David MacLennan's group identified in skeletal muscle sarcoplasmic reticulum (SR) a new Ca^2+^ binding protein and named it the High‐Affinity Ca^2+^ Binding Protein (HACBP).^1^ For a decade, the HACBP did not receive much attention, as it was considered a minor component of the muscle SR membrane, a highly specialized form of endoplasmic reticulum (ER) responsible for handling Ca^2+^ for muscle contraction and relaxation.^2^ Additionally, this protein was difficult to purify and its importance in cellular function or muscle excitation–contraction coupling was not understood. It was in 1980s that I asked, what appears today to be a rather trivial question: what is responsible for Ca^2+^ handling in lumen of the ER in non‐muscle cells? Based on our initial work, it was immediately obvious that HACBP is a major Ca^2+^ binding/storage protein in the lumen of the ER in non‐muscle cells.^3^, ^4^, ^5^, ^6^, ^7^ Because of its Ca^2+^ binding properties and ER luminal localization we chose ‘calreticulin’ as the new name for HACBP.^4^ Isolation of the cDNA for calreticulin and the identification of a C‐terminal KDEL amino acid sequence in the calreticulin protein that serves as an ER retrieval signal brought at that time new excitement to the field of ER cell biology.^4^, ^8^ This coincided with the seminal discovery by the Hugh Pelham group that ER‐resident proteins require a KDEL‐like motif at their extreme C‐terminus to be retained/retrieved to the lumen of ER.^9^, ^10^, ^11^ This firmly established calreticulin as an ER‐resident protein. Today, many more proteins with the KDEL signature have joined calreticulin as ER‐resident proteins.

Ca^2+^ is a universal signalling molecule in control of a spectrum of cellular processes.^12^, ^13^, ^14^, ^15^, ^16^ In the lumen of ER calreticulin binds, stores and delivers Ca^2+^ for many cellular functions.^17^, ^18^, ^19^ It is not surprising therefore that studies on calreticulin biology have revealed the role in or association with many cellular processes: from Ca^2+^ signalling, protein synthesis and folding, posttranslational modification, stress response, energy metabolism, transcriptional regulation, organogenesis, fertilization, viral infection, wound healing, cell–cell communication, immune responses to cell motility and many more. Remarkably, many of these can be explained by the function of calreticulin as a Ca^2+^ handling protein. Numerous review articles have been published on ER, protein quality control, ER chaperones, ER stress and ER Ca^2+^ homeostasis.^12^, ^13^, ^14^, ^15^, ^16^, ^17^, ^19^, ^20^, ^21^, ^22^, ^23^, ^24^, ^25^, ^26^, ^27^, ^28^, ^29^, ^30^, ^31^, ^32^, ^33^, ^34^, ^35^, ^36^, ^37^, ^38^, ^39^, ^40^, ^41^, ^42^, ^43^, ^44^, ^45^, ^46^, ^47^, ^48^, ^49^, ^50^, ^51^ Here, I focus on the biology of calreticulin as a Ca^2+^ binding/handling protein and on the co‐dependence of many Ca^2+^‐dependent cellular processes on Ca^2+^ and calreticulin.

## 2 THE CALRETICULIN GENE

**FIGURE 1:**
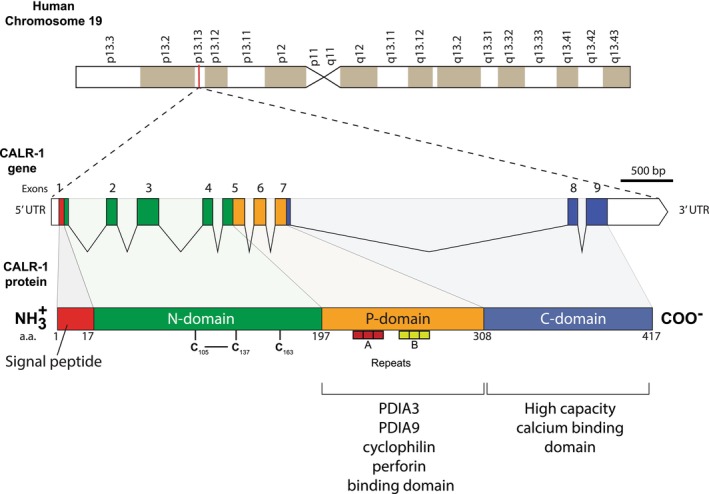
Human calreticulin gene and protein. The human calreticulin gene (*CALR‐1*) is localized to chromosome 19 (19p13.13) and consist of 9 exons over 5.8 kb. The calreticulin protein consists of a signal peptide (red), N‐terminal globular domain (green), central proline‐rich P‐domain (orange) and C‐terminal Ca^2+^ binding C‐domain (blue). Repeats A (amino acid sequence PXXIXDPDAXKPEDWDE [red boxes]) and B (amino acid sequence GXWXPPXIXNPXYX [yellow boxes]) are indicated in the P‐domain. The location of the disulphide bond between Cys105‐Cys137 and free Cys163 in the P‐domain is indicated. CALR, calreticulin; PDI, protein disulphide isomerase; a.a, amino acid.

Two calreticulin genes (calreticulin‐1 and calreticulin‐2) have been identified in human, pig, rat and mouse.^52^, ^53^ Plants have two distinct groups of calreticulin: calreticulin‐1/calreticulin‐2 and calreticulin‐3 group encoded by three calreticulin genes.^37^, ^54^, ^55^ Plant calreticulin genes are not discussed in this review. The calreticulin‐2 gene is expressed specifically in the testis and is silent in other tissues.^52^ The function of *CALR‐2* gene has yet to be determined and it is also not discussed here. The human calreticulin gene (*CALR‐1*) is ubiquitously expressed, consists of 9 exons and spans approximately 5.8 kb and is localized in chromosome 19 (Figure 1).^56^ The nucleotide sequences of the human and mouse genes show greater than 70% identity, indicating a strong evolutionary conservation.^56^, ^57^ Consequently, the regulation of the calreticulin gene, the amino acid sequences and function of the calreticulin protein are also highly conserved. The human and mouse calreticulin promoter contains several binding sites for transcription factors including tissue specific factors. Direct regulation of expression of the calreticulin gene by some of these factors has been confirmed experimentally including Nkx2.5, MEF2C, GATA6, PPARα, PPARγ, COUP‐TF1 and Evi‐1 factors.^58^ The *CALR* gene, along with several other genes, is also activated by environmental stress including depletion of intracellular Ca^2+^ stores in vitro and in vivo.^57^ Increased abundance of calreticulin under environmental stress conditions is a part of the ER stress coping response in attempt to build up ER Ca^2+^ capacity necessary for restoration of ER homeostasis.

Except for their unique C‐terminal domains, calreticulin and calnexin, a type 1 integral ER membrane chaperone (discussed below), share many structural features.^17^, ^59^ However, the human calnexin gene (*CANX*) consist of 15 exons (33 kb) encoding 592 amino acid residue polypeptide and it is located on chromosome 5.^59^, ^60^ Furthermore, phylogenetic analysis indicates that calreticulin and calnexin existed as separate proteins derived from independent clades.^61^ It is important to realize that calnexin is anchored to the ER membrane by transmembrane domain with limited mobility in the ER^59^ whereas calreticulin is a resident ER luminal protein with high Ca^2+^ binding capacity free to move in the ER lumen.^17^

## 3 THE CALRETICULIN PROTEIN

**FIGURE 2:**
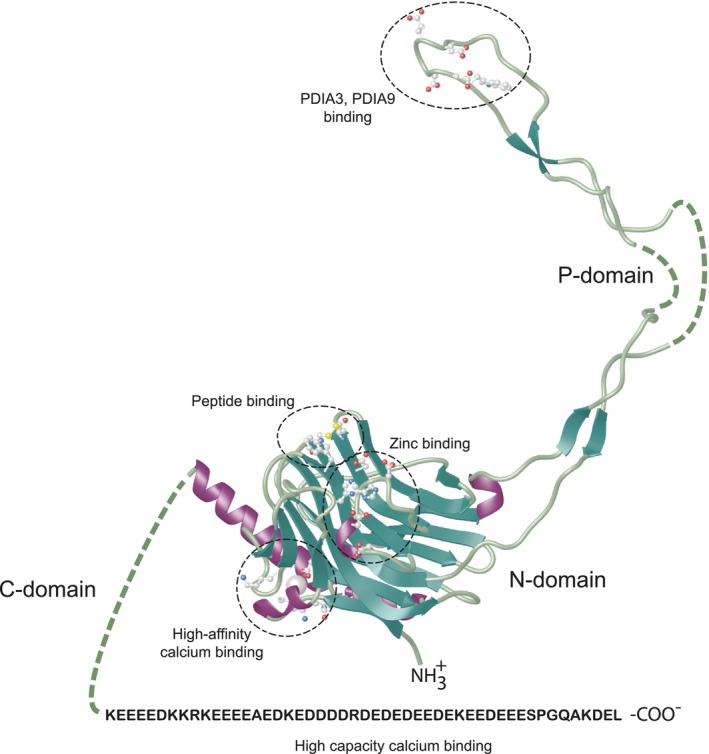
Three‐dimensional structure of calreticulin. The crystal structure of the human calreticulin CALR‐1. The location of peptide binding, the high‐affinity Ca^2+^ binding site and Zn^2+^ binding site is indicated. The PDIA3 and PDIA9 binding site is located at the tip of the P‐domain. A stretch of acidic amino acid residues in the C‐domain is involved in a high‐capacity Ca^2+^ binding is shown. The broken line represents unstructured regions of the protein. The model is based on X‐ray, Cryo‐EM and NMR data: PDB ID:1HHN, PDB ID:3POW.^38^, ^62^, ^63^, ^64^, ^65^

Calreticulin is made up of 417 amino acids, including a 17 residue N‐terminal signal sequence directing the protein to the ER and a C‐terminal KDEL ER retrieval signal (Figures 1 and 2). The protein is composed of three structural and functional domains. These domains were originally defined based on computational analysis of the amino acid sequence,^4^, ^17^ but subsequently confirmed and refined based on the empirical (mutational and structural) analysis of the purified protein (Figures 1 and 2).^62^, ^63^, ^64^, ^66^, ^67^, ^68^, ^69^, ^70^, ^71^, ^72^, ^73^

The highly conserved globular N‐terminal domain contributes to carbohydrate and polypeptide binding, and has binding sites for Zn^2+^ (Figure 2).^68^, ^73^, ^74^, ^75^ Many chaperones are regulated by ATP to facilitate chaperone–client interactions.^76^, ^77^ As such, ATP also binds to calreticulin^78^, ^79^, ^80^ and calnexin,^81^, ^82^ resulting in conformational changes.

The P‐domain of calreticulin forms an extended arm which contains a binding site for the thiol oxidoreductase PDIA3 (also known as ERp57),^83^, ^84^ PDIA9 (also known as ERp29)^85^ and cyclophilin B (Figure 2).^86^, ^87^ The interaction between calreticulin and PDIA3 supports protein folding in the ER.^20^ The P‐domain also interacts with perforin, a pore forming protein in the granules of cytotoxic T lymphocytes.^88^, ^89^, ^90^ Originally, the N‐ and P‐domains were considered as the core of the chaperone unit of calreticulin.^91^ Structural analyses of calreticulin by Kalle Gehring's group showed that a part of the C‐domain (from P^301^ to E^363^) also contributes to a carbohydrate binding pocket of the protein.^38^, ^62^ Recombinant calreticulin P‐domain purified from *E. coli* binds Ca^2+^ with high affinity (*K*_d_ < 1 μM) but at low capacity (1 mol of Ca^2+^/mol of protein).^60^, ^64^, ^91^, ^92^ Analysis of the crystal structures of the N‐ and P‐domains of calreticulin identified a Ca^2+^ ion coordinated by amino acids Asp328, Gln26, Lys62 and Lys64^62^ corresponding to the high‐affinity Ca^2+^ binding side identified for calreticulin. This Ca^2+^ remains tightly bound to the protein and coordinates the calreticulin structure. The loss of Ca^2+^ binding to the high‐affinity site is expected to result in a drastic conformational change in calreticulin.

The C‐domain of calreticulin, which contains five clusters of acidic amino acid residues, is the Ca^2+^ binding unit of the protein and binds Ca^2+^ with low affinity (*K*_d_ = 2 mM) but at high capacity (20–30 mol of Ca^2+^/mol of protein).^60^, ^91^ The unstructured C‐terminal Ca^2+^ binding domain terminates with a KDEL amino acid sequence that is responsible for ER retrieval of the protein. The C‐domain of calreticulin is responsible for maintaining 50% of the total cellular Ca^2+^ within the ER.^91^, ^93^ Mutations in exon 9, encoding the C‐domain of the protein, were identified in myeloproliferative neoplasms (MPNs), essential thrombocythaemia, and primary myelofibrosis resulting in a frameshift within exon 9 to generate a novel C‐terminal amino acid sequence.^94^, ^95^, ^96^ The beauty of calreticulin is that each of the calreticulin domains perform specialized Ca^2+^‐dependent functions in the ER: the N‐ and P‐domains playing a role of lectin‐like chaperone, the P‐domain providing site for Ca^2+^‐dependent docking of folding enzymes and the C‐domain being necessary for the high‐capacity Ca^2+^ binding role of the protein (discussed below).

### 3.1 Three‐dimensional structure of calreticulin

One important advance in our understanding of the structure of calreticulin was the elucidation of the crystal structure of the luminal domain of calnexin. Calnexin shares similarity with calreticulin's amino acid sequence encoding N‐ and P‐domains.^59^, ^73^, ^97^, ^98^ Combined with solving the structure of the P‐domain of calreticulin by NMR technique,^64^ the 3D structure provided for the first‐time structural information on calreticulin (reviewed in^38^).

For a decade, many attempts were undertaken to obtain a crystal structure of calreticulin. These efforts failed likely due to the flexible nature of the extended P‐domain arm and unstructured C‐domain. A major breakthrough came from Kalle Gehring's group who was successful in solving the three‐dimensional structure of recombinant calreticulin expressed without a larger portion of the flexible C‐domain.^38^, ^62^ This was followed by determination of the crystal structure of the globular domain of the calreticulin^63^ and Cryo‐EM studies of the human MHC‐I peptide loading complex.^65^ A large portion of the C‐domain (>30 residues) remains missing in these structures due to being highly disordered. Isolated C‐domain of calreticulin remains disorder at low Ca^2+^ concentration but shows a more compact conformation at the high Ca^2+^ concentration.^99^, ^100^ This likely contributes to a Ca^2+^ sensor‐like function of calreticulin.^99^ Recent elegant studies on structural properties of calreticulin mutants associated with MNP indicate that the C‐domain is partially α‐helical and it plays a role in CALRdel52 mutant interaction with the TpoR.^101^ Considering the high concentration of calreticulin in the ER lumen, the highly flexible (and mobile) Ca^2+^ binding C‐domain of calreticulin and extended P‐domain arm of the protein are designed to protect the ER luminal environment against self‐aggregation (crystallization) of the protein. Ca^2+^ binding to calreticulin also contributes to preventing protein aggregation by prompting conformational changes sensitive to ER Ca^2+^ fluctuations.^102^ The presence of the highly flexible protein regions and the unstructured low‐affinity Ca^2+^ binding segments are likely a common protective mechanism for preventing self‐aggregation of other ER‐resident proteins that are highly concentrated in the ER lumen (100 mg protein/ml representing over 3% of all human proteins [Human Protein Atlas; https://www.proteinatlas.org/humanproteome/subcellular/endoplasmic+reticulum#:~:text=246%20proteins%20in%20the%20endoplasmic,a%20cell%20to%20cell%20variation]).

Based on available structural information, it is now possible to build a three‐dimensional model of calreticulin with recognizable functionally important regions and identification of specific amino acid residues necessary for these functions (Figure 2). The carbohydrate recognition site encompasses amino acid residues 18–203 in the N‐domain and residues Pro301 to Glu363 in the C‐domain.^38^, ^62^ Site specific mutation analysis also identified a disulphide bridge between Cys105 and Cys137, and Lys111 as important in the binding of carbohydrate moieties by calreticulin.^38^, ^62^, ^75^ Notably, substitution of Cys137Ala, Cys105Ala and Trp319Ala alter chaperone function in vitro.^66^ These mutations change the flexibility of the calreticulin backbone and the secondary structure of the N‐domain due to new interdomain contacts between the P‐domain, globular N‐domain and parts of the C‐domain.^70^ The Lys111Ala substitution impairs calreticulin–carbohydrate interactions,^75^ and Cys105 and Cys137 are involved in contacts with carbohydrate.^62^ Cys105, Cys137, Asp135 and Trp319 were identified important in peptide binding to calreticulin.^63^ Although four His residues within the N‐domain of calreticulin have been proposed to coordinate zinc binding,^103^, ^104^, ^105^ only His42 is accessible and together with Asp118, Asp121, His123 and Asp125 it forms a zinc binding site.^62^ However, the importance of these residues in the binding of zinc needs to be experimentally tested. Biochemical and biophysical analyses of several calreticulin mutants identified additional structural features of the protein, including conserved clusters of surface exposed amino acid residues important for maintaining structural stability of the protein.^71^ Tyr172 and Asp187 are crucial for maintaining the native structure of the protein, while Tyr172 engages the free Cys163 residue to support the thermal stability of calreticulin.^71^

Amino acid residues Glu255, Glu256, Asp248, Glu260, Trp261 (Figure 2) at the tip of the hairpin‐like structure of the P‐domain are the site of binding of PDIA3, a folding enzyme^66^, ^67^, ^68^, ^69^ and PDIA9, a protein involved biosynthesis and trafficking of secretory and transmembrane proteins (Figure 2).^106^, ^107^ Cyclophilin B, a member of the family of peptidyl cis/trans protein isomerases (PPI), important for collagen folding,^108^ also binds to the tip of the P‐domain of calreticulin (Figure 2).^85^ PDIA1 was the first oxidoreductase identified as interacting with calreticulin^109^ and PDIA1 binding to calreticulin affects luminal ER redox conditions upon Ca^2+^ depletion,^110^ linking ER luminal redox environment to Ca^2+^ handling. Being a member of the oxidoreductases family of folding enzymes it is likely that PDIA1 also docks on the tip of the P‐domain. Most importantly, under physiological conditions interactions between the P‐domain of calreticulin and oxidoreductases are sensitive to ER luminal Ca^2+^ provided by calreticulin.

Considering the three‐dimensional arrangement of the P‐domain as a long arm extending from the globular, carbohydrate binding domain of calreticulin, the tip of the P‐domain offers a perfect docking place for folding enzymes to associate with calreticulin to accelerate protein folding. The breakthrough advances in structural studies of calreticulin revealed a distinctive 3D architecture of the protein domains and helped to understand how calreticulin domains perform Ca^2+^‐dependent specialized functions; from Ca^2+^ binding, storage and control of Ca^2+^‐dependent interactions with folding enzymes to lectin‐like chaperone function.

### 3.2 Phenotypes associated with known calreticulin gene variants

**FIGURE 3:**
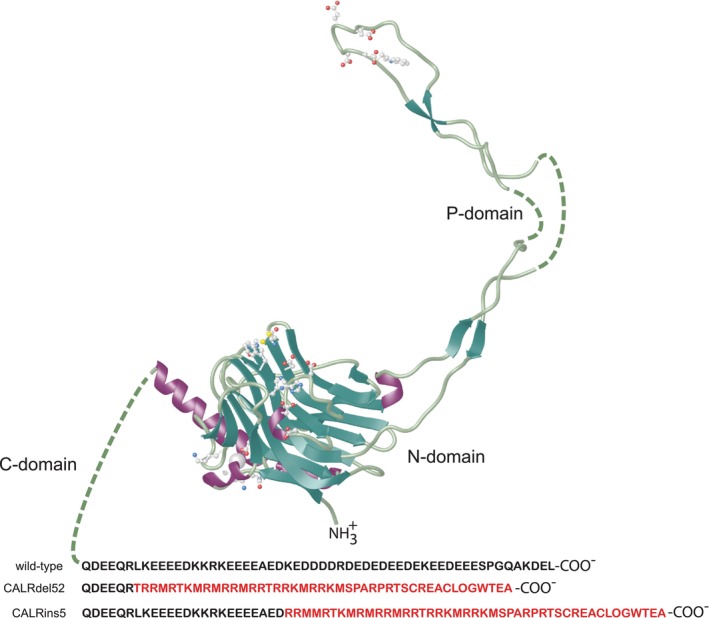
Model of calreticulin variants identified in myeloproliferative neoplasm. The frameshift within the exon 9 of the calreticulin gene generates a novel C‐terminus with most of negatively charges amino acid residues of the native C‐domain replaced with positively charged amino acid residue. The two most common calreticulin mutations are shown in the Figure: a 52 base pair deletion (*CALRdel52*) and a 5 base pair insertion (*CALRins5*). These mutations generate novel amino acid sequence of the C‐domain (shown in red). The ER retrieval KDEL sequence is lost in both mutants. Amino acid sequence of the native C‐domain (*wild‐type*) is also shown.

Recently, there has been a renewed interest in calreticulin due to a discovery of calreticulin gene variants associated with chronic blood tumours known as myeloproliferative neoplasm (MPN).^94^, ^95^, ^96^ More than 60 different variants leading to a shift of the reading frame in calreticulin exon 9 (encoding the Ca^2+^ binding C‐domain of the protein) have been identified in more than 30% of MPN patients.^94^, ^95^, ^111^, ^112^ The two most common variants are a 52 base pair deletion (Type 1/*CALR*del52 frequency 53%) and a 5 base pair insertion (Type 2/*CALR*ins5 frequency 32%). These shift of the reading frame mutations result in expression of a novel C‐terminal amino acid in the calreticulin protein (Figure 3). Both frameshift mutations cause a loss of KDEL ER retrieval sequence with some of mutant calreticulin localized to the ER of haematopoietic cells and remaining being transported out of the ER and mis‐localized to the cell surface.^113^, ^114^, ^115^, ^116^, ^117^ On cell surface, mis‐localized calreticulin variants bind to the thrombopoietin receptor (TpoR), and activate downstream JAK2/STAT5/STAT3 signalling.^114^, ^117^, ^118^, ^119^, ^120^, ^121^ Furthermore, calreticulin mutants secreted from malignant cells inhibit phagocytosis of dying cancer cells by dendritic cells resulting in immunosuppressive effects.^122^ Importantly, the frameshift mutations of calreticulin render the C‐terminus of the protein with positively charged, affecting the Ca^2+^ capacity to the protein.^115^ Comparison of CALRdel52 to CALRins5 (Figure 3) indicates that while CALRdel52 has all the negative charges of the calreticulin C‐terminus converted to positive charges, there is only a ~ 50% change in charge in the CALRins5 variant.^94^, ^95^, ^112^ The loss of Ca^2+^ binding sites on the CALRdel52 mutant (not to CALRins5) results in reduced ER Ca^2+^ storage capacity and activation of UPR.^123^

There are also a numerous somatic mutations in the calreticulin gene identified in variety of cancers (https://www.cbioportal.org/results/mutations?case_set_id=all&gene_list=CALR&cancer_study_list=5c8a7d55e4b046111fee2296). These mutations are scattered across all regions of the protein but their functional consequences and their role in cancer biology are not known.

**FIGURE 4:**
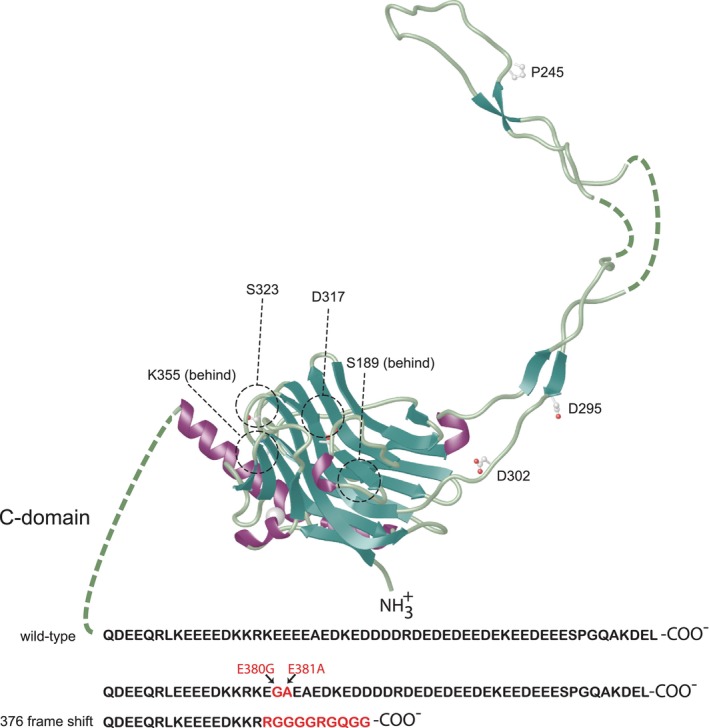
Model of calreticulin depicting protein variants identified in sudden death patients. Shown is the location of calreticulin mutations identified in sudden unexpected death patients. A direct link between mutations of the calreticulin gene and sudden death has not been established. Information on possible related mutations was received from Drs. D.J. Tester and M.J. Ackerman at the Windland Smith Rice Sudden Death Genomics Laboratory at the Mayo Clinic in Rochester, Minnesota.

Recently, several variants in the calreticulin gene have been identified in sudden unexpected death patients^71^ (Figure 4) although their contribution to the sudden unexpected death remains to be established. Intriguingly, these mutations are identified in different regions of calreticulin including the globular N‐domain, P‐domain and Ca^2+^ binding C‐terminal domain (Figure 4). These mutations are predicted to affect protein stability and Ca^2+^ binding. Sudden unexpected death is frequently associated with heart failure due to altered Ca^2+^ handling by cardiomyocytes^124^, ^125^, ^126^ and impaired Ca^2+^ binding to calreticulin variants is expected to contribute to the sudden unexpected death phenotype (see below for a role of calreticulin Ca^2+^ pool in cardiac pathophysiology).

## 4 THE ENDOPLASMIC RETICULUM: CALRETICULIN'S ‘DOMICILE’

The ER is a multifaceted and multifunctional cellular organelle.^19^, ^34^, ^51^, ^127^, ^128^, ^129^, ^130^, ^131^ The ER is a major component of the cellular reticular network (CRN) that includes the Golgi apparatus, lysosomes, peroxisomes, components of the endocytic pathway, as well as the nuclear envelope,^19^, ^34^, ^128^ and occupies about 50% of the total CRN membrane area.^19^, ^34^, ^128^ The ER is the site for the synthesis of many proteins and lipids, structural components of biological membranes. Furthermore, the ER forms functional connections (membrane contact sites) with other cellular organelles, and the plasma membrane impacting on organellar and cell–cell communication.^12^, ^19^, ^34^, ^51^, ^127^, ^132^, ^133^, ^134^, ^135^, ^136^, ^137^ Considering the dynamic connections between the ER and other intracellular organelles within the CRN,^19^, ^34^, ^128^, ^138^, ^139^ it is not surprising that calreticulin has been detected in other organelles including Golgi, endosomes, cytolytic granules and cortical granules.^88^, ^140^, ^141^, ^142^, ^143^, ^144^ Consequently, both calreticulin and Ca^2+^, being centrally located in the ER, have the ability to control major cellular functions far beyond the ER.

The main intracellular site for Ca^2+^ storage and signalling is the ER.^12^ ER luminal Ca^2+^ modulates many ER functions including protein synthesis and folding, protein quality control, chaperone–chaperone/folding enzymes and chaperone–client interactions, and ER stress coping responses including the unfolded protein response (UPR).^12^ In addition to the high content of Ca^2+^, the ER is well equipped with many resident proteins that monitor and assess cellular needs, respond to changes in cellular and organellar homeostasis, perform adjustments to homeostasis, eliminate misfolded proteins and maintain its own healthy homeostasis for immediate stress/emergency responses. Just like a hospital ‘emergency room (ER)’, the ER with its extraordinarily wide spectrum of associated functions and the ability to deal with many cellular emergencies. Calreticulin lives in the crowded ER ‘emergency room’ where it has the responsibility of supplying and managing Ca^2+^‐dependent events.

There are instances when calreticulin is found outside the cell (reviewed in^30^). For example, extracellular calreticulin has been seen to bind to thrombospondin and participate in cell signalling leading to the assembly and disassembly of focal adhesions and increased motility.^145^, ^146^, ^147^ Leslie Gold's group discovered that extracellular calreticulin promotes repair of cutaneous wounds.^30^, ^148^, ^149^, ^150^, ^151^, ^152^ Extracellular calreticulin plays a protective role in the central nervous system as brain cells release native calreticulin under ER stress conditions.^153^, ^154^ Furthermore, extracellular calreticulin (or the C‐domain fragment of the protein) binds to blood‐clotting factors and inhibits injury‐induced restenosis.^155^

Calreticulin has been linked to many cancers (reviewed by Fucikova et al.^28^). The Peter Henson group^156^ discovered that extracellular calreticulin binds to and activates the LDL receptor‐related protein (LRP) on phagocytic cell to prevent activation of the ‘don't eat me’ signal. Obeid et al.^157^ reported that in mice extracellular calreticulin generates ‘eat me’ signal for phagocytic cells. Extracellular calreticulin has also been identified as pro‐phagocytic signal on the surface of several human cancers as well as vital for the immunogenic cell death of cancer cells.^158^, ^159^, ^160^ Thus, extracellular calreticulin promotes the uptake of cell by professional phagocytes and initiates of anticancer immunity. Many years ago, it was found that individuals infected with *Trypanosoma cruzi* presented with a low incidence of cancer compared to non‐infected individuals.^161^ This was many years before calreticulin was discovered. Today it is known that calreticulin secreted by *T. cruzi* plays a protective role in cancer by increasing tumour immunogenicity and inhibiting tumour growth.^162^, ^163^, ^164^, ^165^, ^166^, ^167^, ^168^, ^169^ Both *T. cruzi* calreticulin and host extracellular calreticulin likely affect the immune response in cancer by similar mechanisms. The pool of calreticulin (ER versus extracellular and/or Ca^2+^ handling by calreticulin) responsible for the beneficial or detrimental role in cancer remains to be determined.

Importantly, it remains to be established whether and how calreticulin finds a way to exit the ER and travels all the way to the cell surface to influence pathophysiology. This may be facilitated by various stress stimuli, such as inflammation and hypoxia^45^ or release of calreticulin from broken or apoptotic cells. The protein may also leave the ER bound to secreted proteins or newly synthesized cell surface receptors.^170^, ^171^ Regardless, it is inevitable that the departure of calreticulin from the ER (its domicile) alters cellular Ca^2+^ handling.

## 5 CALRETICULIN, THE CHAPERONE

**FIGURE 5:**
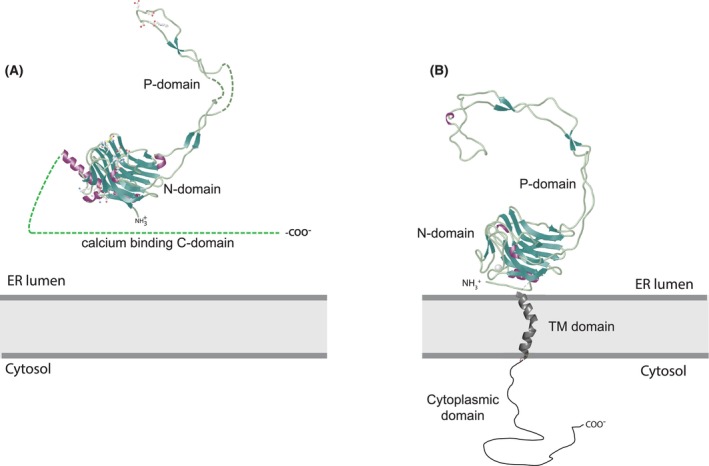
Calreticulin and calnexin. Calreticulin (A) and calnexin (B) share amino acid sequence similarities in their N‐terminal globular N‐domain and proline‐rich P‐domain. Calreticulin is an ER‐resident soluble protein (A) whereas calnexin is a type 1 integral ER membrane protein (B). The C‐terminal regions of the proteins are unique for each protein. Calreticulin has a high‐capacity Ca^2+^ binding C‐domain whereas C‐terminal domain of calnexin is extended into cytosol. Calreticulin model is based on PDB ID:1HHN, PDB ID:3POW. Calnexin model based on PDB ID:1JHN. TM, transmembrane.

The role of calreticulin as a molecular chaperone and a component of the calreticulin/calnexin cycle has been extensively reviewed.^20^, ^21^, ^22^, ^39^, ^172^, ^173^, ^174^, ^175^, ^176^, ^177^, ^178^, ^179^ The calreticulin/calnexin cycle is central to the protein quality control machinery, which disposes of misfolded secretory proteins before they can exit the ER.^20^, ^180^, ^181^ Since calreticulin is an ER lumen‐resident protein, it is unconstrained and can move freely within the ER lumen. In contrast calnexin movement is restricted in the ER because it is an integral ER membrane protein anchored to the membrane via a transmembrane helix followed by large cytoplasmic domain (Figure 5).^59^ The chaperone function of calreticulin and calnexin ER luminal domain is interchangeable but the spectrum of glycoproteins that calreticulin or calnexin bind is determined by topological environment, that is whether they are attached to a membrane (calnexin) or are free in the ER lumen (calreticulin).^182^ Regardless, both proteins require the presence of Ca^2+^ (supplied by calreticulin) to perform their role of chaperones.^60^, ^66^

The role of calreticulin in the immune system has been extensively studied.^20^, ^173^, ^183^, ^184^, ^185^, ^186^, ^187^, ^188^, ^189^ The protein plays a role in the assembly and maturation of MHC‐I into the protein–peptide complex and the generation of effective cytotoxic T‐cell responses.^183^, ^184^, ^185^ Calreticulin forms heterodimeric complexes with PDIA3, a thiol oxidoreductase, and together they assist in the biogenesis of MHC class I. Calreticulin is also a structural component of the peptide loading complex, which consists of the HC‐β2m heterodimer, calreticulin, and the additional components tapasin, transporter associated with antigen processing (TAP), and Bap31.^173^, ^186^, ^187^ TAP provides a major source of peptides for MHC class I molecules, whereas tapasin, PDIA3, and calreticulin facilitate assembly of MHC class I molecules with peptides.^190^ These important processes are Ca^2+^ sensitive and they depend on the presence of ER luminal Ca^2+^ supplied by calreticulin. Not surprisingly, MPN calreticulin C‐domain deletion mutants are functionally impaired in facilitating MHC‐I Class II or Class I assembly^188^, ^189^ and their interaction with PDIA3 and tapasin is also reduced.^188^

## 6 CALRETICULIN, THE CA^2+^ HANDLING PROTEIN

Ca^2+^ is an important signalling molecule that has huge influence over nearly all cellular functions including gene expression, stress coping responses, motility, cell adhesion, muscle contraction, protein secretion, proliferation, apoptosis, cell metabolism and fertilization.^12^, ^13^ The success or failure to control Ca^2+^ homeostatic/signalling mechanisms have life or death consequences for the cell and the organism. Formation of intracellular Ca^2+^ stores, the source of immediately available Ca^2+^ to facilitate Ca^2+^ signalling and Ca^2+^‐dependent communication between intracellular organelles, is a decisive advantage making cells less dependent of the extracellular Ca^2+^. In the ER, calreticulin is intimately involved in integrating and coordinating many Ca^2+^‐dependent pathways in virtually all cellular compartments.^137^, ^191^ What distinguishes various cells and tissues are the specific mechanisms that regulate the initial extracellular Ca^2+^ influx and the intracellular Ca^2+^ mobilization. Importantly, these mechanisms are dependent on Ca^2+^ stores and Ca^2+^ storage/binding proteins. Calreticulin functions as a Ca^2+^ storage protein and, in turn, Ca^2+^ is dependent on calreticulin for maximal retention in the ER against continues flow of molecules and ions down the secretory pathway. Sometimes calreticulin can be found in intracellular organelles other than the ER where it manages Ca^2+^ to support specialized functions and heterogeneity of the CRN.

### 6.1 ER and Ca^*2*+^ import, export and storage

Even though the total ER intraluminal Ca^2+^ concentration (includes free Ca^2+^ and Ca^2+^ bound to ER luminal resident proteins) is in excess of 2 mM, the free Ca^2+^ concentration is maintained within the 100–800 μM range but could increase to as high as 1000 μM under certain conditions.^191^, ^192^, ^193^, ^194^, ^195^, ^196^ The wide range of free ER luminal Ca^2+^ concentrations is interpreted as being due to methodological challenges associated with measuring organellar ion concentrations. However, it is also possible that this reflects the dynamic nature of Ca^2+^ concentration changes in the ER lumen driven by cellular Ca^2+^ needs and environmental conditions. The total Ca^2+^ concentration in the cytoplasm is also high (mM range), but in contrast to the ER lumen, the cytoplasmic free Ca^2+^ is maintained at <100 nM.^197^ Once released from calreticulin, Ca^2+^ can move much easier in the ER lumen,^198^, ^199^ but in the cytosol Ca^2+^ is maintained under strict control, where it is bound to many high‐affinity Ca^2+^ binding proteins and cytoskeletal components. In the cytosol Ca^2+^ functions as universal signalling molecule that regulates many cellular processes, including cell proliferation, metabolism and apoptosis.^51^, ^197^ In the lumen of ER, Ca^2+^ also plays a signalling role in regulating protein synthesis, folding, posttranslational modification and trafficking.^109^, ^191^, ^200^, ^201^

**FIGURE 6:**
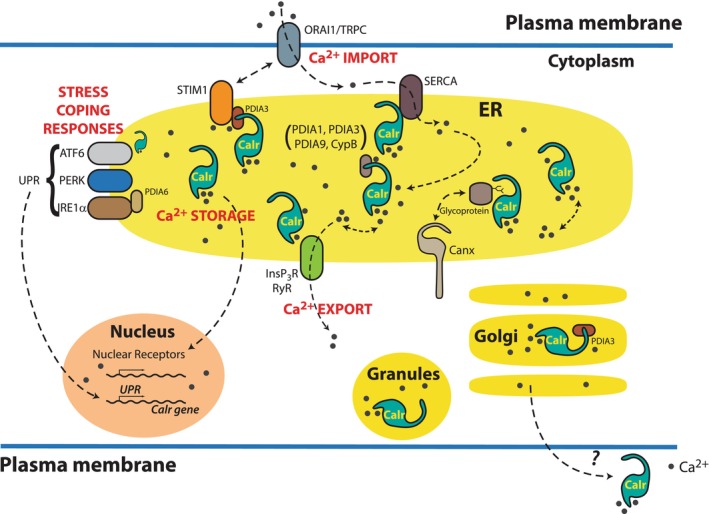
ER, calreticulin's ‘domicile’. Calreticulin is an ER‐resident Ca^2+^ binding/storage protein. Ca^2+^ can be easily and rapidly released from calreticulin to other Ca^2+^ handling proteins. Ca^2+^ managed by calreticulin plays a signalling role and supports many protein–protein, chaperone–client interactions, as well as impacting stress responses and regulation of the UPR (stress coping response). In cells STIM1/ORAI1/TRPC together with SERCA are responsible for Ca^2+^ import to the ER whereas InsP_3_R and RyR play a role in Ca^2+^ export from the ER. There are cases when calreticulin can be found outside the ER but how calreticulin exits the ER remains to be established. ATF6, activating transcription factor 6; Calr, calreticulin; Canx, calnexin; CypB, peptidylprolyl isomerase B; ER, endoplasmic reticulum; InsP_3_R, inositol trisphosphate receptor/Ca^2+^ channel; IRE1α, inositol‐requiring enzyme 1α; ORAI1, Ca^2+^ release‐activated Ca^2+^ channel protein 1; PERK, protein kinase RNA‐like endoplasmic reticulum kinase; PDI, protein disulphide isomerase RyR, ryanodine receptor/Ca^2+^ channel; SERCA; sarcoplasmic/endoplasmic reticulum Ca^2+^‐ATPase; SOCE, store‐operated Ca^2+^ entry; STIM1, stromal interaction molecule 1; UPR, unfolded protein response; TRPC, transient receptor potential channel.

Once accessed from calreticulin, Ca^2+^ is released from the ER (*ER Ca* ^*2+*^ *export*) via the inositol 1,4,5‐trisphosphate receptor (InsP_3_R)/Ca^2+^ channel or/and the ryanodine receptor/Ca^2+^ channel (RyR) (Figure 6).^12^, ^13^, ^202^, ^203^ The depletion of ER Ca^2+^ triggers Ca^2+^ entry from the extracellular space by store‐operated Ca^2+^ entry (SOCE), which plays a major role supplying Ca^2+^ for refilling of the ER Ca^2+^ store (*ER Ca* ^*2+*^ *import*) (Figure 6).^12^, ^192^, ^204^, ^205^ Stromal interaction molecule 1 (STIM1) is an ER membrane type 1 transmembrane protein that responds to reduced ER luminal Ca^2+^ concentration.^204^, ^205^, ^206^, ^207^, ^208^ In the plasma membrane the two main Ca^2+^ influx channels are the ORAI and transient receptor potential channels (TRPC) that function in a STIM1‐dependent way.^209^ Upon ER Ca^2+^ depletion (dissociation of Ca^2+^ from calreticulin), STIM1 clusters move towards the plasma membrane containing ORAI1 and TRPC and form membrane contacts to activate SOCE.^139^, ^205^, ^208^, ^209^ The sarco‐endoplasmic reticulum Ca^2+^ ATPase (SERCA) pumps Ca^2+^ from the cytosol into the ER for storage bound to calreticulin (*ER Ca* ^*2+*^ *import*) (Figure 6).^12^, ^13^ The calreticulin mutant expressed in MPN cells causes abnormal regulation of SOCE^206^ accounting for dysregulated Ca^2+^ fluxes in MPN cells.^119^ It is not surprising cells expressing MPN variants of calreticulin have increased cytosolic Ca^2+^ concentrations.^111^

Over 50% of Ca^2+^ stored in the ER is bound to calreticulin (*ER Ca* ^*2+*^ *storage*).^18^, ^34^, ^93^, ^210^ In addition, the ER lumen contains other Ca^2+^ binding‐resident chaperones (GRP94, BiP/GRP78) and folding enzymes^18^, ^19^ but these proteins cannot substitute for calreticulin as a Ca^2+^source as they bind less Ca^2+^ and are engaged in other specialized activities in the ER. However, calreticulin can hold large amounts of Ca^2+^ (‘calreticulin Ca^2+^ pool’) and depending on cellular needs Ca^2+^ can be easily and rapidly released from the protein to other Ca^2+^ handling proteins making calreticulin an ideal protein for regulating the activities of Ca^2+^‐dependent cellular processes.^18^, ^19^, ^211^, ^212^ For example, increase in the abundance of calreticulin Ca^2+^ pool is vital for repetitive InsP_3_‐induced Ca^2+^ waves (Ca^2+^ export).^213^ Calreticulin also regulates SERCA2b function (Ca^2+^ import) either directly^214^ or via complex formation with PDIA3.^215^ Increased expression of calreticulin (increased calreticulin ER Ca^2+^ pool) has inhibitory effect on SOCE (Ca^2+^ import).^216^, ^217^ Furthermore, Ca^2+^ entry from the extracellular space via SOCE (Ca^2+^ import) is modulated by PDIA3 binding to STIM1^218^, ^219^ and calreticulin interacts with PDIA3 in a Ca^2+^‐ and calreticulin conformation‐dependent manner.^102^, ^200^ Importantly, depending on the environmental conditions, Ca^2+^ is released from calreticulin when the protein undergoes conformational changes and ‘senses’ fluctuations in the ER luminal Ca^2+^ concentration due to active Ca^2+^ export/import and/or when calreticulin interacts with client proteins, chaperones, folding enzymes or Ca^2+^ handling proteins such as InsP_3_R, STIM1 or SERCA.

The ER is heterogeneous with respect to the distribution of Ca^2+^ handling proteins (Ca^2+^ pumps, Ca^2+^ release channels)^220^ and calreticulin delivers Ca^2+^ needed to maintain the functional heterogeneity of the ER Ca^2+^ access to Ca^2+^ handling proteins and organelles in the CRN. Finally, by handling the ER luminal Ca^2+^, calreticulin prevents Ca^2+^ misuse that results in altered cell shape, cell motility, cell proliferation, membrane damage, mitochondrial calcification, impaired energy metabolism, improper folding of proteins and cell death.

### 6.2 Calreticulin, Ca^*2*+^ and ER stress

The ER plays a central role in managing cellular stress via mobilization of ER stress‐coping responses, such as the UPR. The UPR is comprised of three signalling arms controlled by ER‐associated integral membrane stress sensor proteins: the ER kinase dsRNA‐activated protein kinase‐like ER kinase (PERK), activating transcription factor 6 (ATF6) and the serine/threonine‐protein kinase/endoribonuclease inositol‐requiring enzyme 1α (IRE1α) (Figure 6). These stress sensors bind the ER lumen chaperone BiP/GRP78 and under stress conditions BiP/GRP78 dissociates from IRE1α, PERK and ATF6 resulting in activation of UPR. There are two isoforms of ATF6, ATF6α and ATF6β, with ATF6α playing a primary role in the UPR. Interestingly, calreticulin gene is a target of ATF6β in the central nervous system.^221^, ^222^ The ATF6β‐dependent regulation of expression of calreticulin plays crucial role in neuronal survival under ER stress by improving intracellular Ca^2+^ homeostasis.^221^, ^222^ This further supports a notion that UPR signalling is designed to increase the expression of protein chaperones including calreticulin resulting in increased ER Ca^2+^ capacity.^57^, ^221^, ^222^ Concomitant with this is increase in a number of ER‐mitochondrial contact sites,^223^, ^224^ promoting transfer of Ca^2+^ accumulated in the ER due to increase in calreticulin Ca^2+^ pool, to mitochondria in exchange for ATP.^223^, ^224^ Calreticulin role in maintaining high ER Ca^2+^ capacity is, therefore, vital in supporting energy production especially during times of cellular stress. Upregulation of the calreticulin gene expression at the time of cellular stress is a clever strategy to maintain high ER Ca^2+^ capacity which is central for successful UPR coping responses.

### 6.3 Calreticulin versus calsequestrin

**FIGURE 7:**
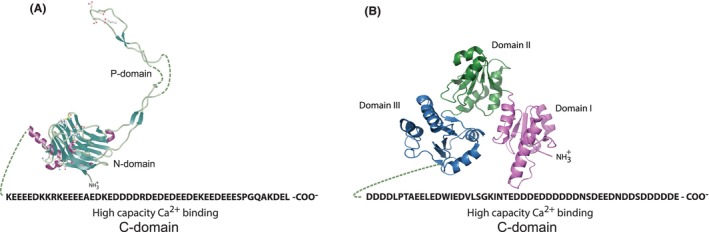
Calreticulin and calsequestrin. Calreticulin (A) and calsequestrin (Casq2) (B) are major Ca^2+^ binding proteins in the lumen of the ER and the junctional SR, respectively. The proteins share amino acid sequence similarities of their unstructured and flexible C‐terminal domains responsible for high‐capacity Ca^2+^ binding. Three thioredoxin fold domains of calsequestrin are indicated in the Figure (domains I, II, III). The N‐terminal regions of calreticulin (A) and calsequestrin (B) are unique for each protein. Calreticulin model is based on PDB ID:1HHN, PDB ID:3POW. Calsequestrin model based on PDB ID:2VAF.

In the skeletal and cardiac muscle, the majority of the ER membrane is organized into highly specialized membrane network referred to as sarcoplasmic reticulum (SR) responsible for handling Ca^2+^ for the muscle contraction and relaxation. Functionally, muscle cells, just like any other cell, also contain conventional ER membranes equipped with and responsible for common cellular functions.^2^ In muscle SR, calsequestrin is the major Ca^2+^ binding/storage protein, and is localized to the junctional SR where it functions as the source of Ca^2+^ for released by RyR to trigger muscle contraction.^202^, ^218^, ^225^, ^226^, ^227^ There are two isoforms of calsequestrin encoded by different genes, namely *CASQ1* and *CASQ2*.^225^ The N‐terminal region of calsequestrin contains three domains with high structural similarity to the thioredoxin fold found in PDI‐like proteins (Figure 7),^225^, ^228^ and is distinct from the 3D structure of the N + P‐domain of calreticulin (Figure 7). Although the N‐terminal domains of calreticulin and calsequestrin are unique to each protein, both calreticulin and calsequestrin share amino acid sequence similarities in their C‐terminal domains responsible for high‐capacity Ca^2+^ binding (Figure 7). Calsequestrin is designed to store Ca^2+^ in the junctional SR and plays a limited role confined to support of muscle excitation–contraction coupling.^225^ The protein localization in muscle cells is restricted to the junctional SR due to oligomerization^225^, ^229^, ^230^ and binding to the junctional SR proteins including RyR, triadin, junctin, IRE1α.^225^ In contrast, calreticulin is strategically positioned in the lumen of the ER in all cells (including muscle cells) from where the protein manages access to and distribution of Ca^2+^ for not just one but many cellular Ca^2+^‐signalling events. Thus, muscle cells differ from many cells because they express two high‐capacity Ca^2+^ binding proteins (calreticulin and calsequestrin) and, consequently, muscle cells contain two large and distinct pools of organellar Ca^2+^: calreticulin Ca^2+^ pool and calsequestrin Ca^2+^ pool (discussed below).

## 7 CALRETICULIN CA^2+^ POOL: LESSONS FROM THE HEART

Another major advance in calreticulin research was the creation of the calreticulin‐deficient mouse model.^2^, ^93^ This was the first animal model with targeted inactivation of a gene encoding an ER luminal protein. In mice, calreticulin deficiency is lethal at embryonic day 18.5 due to impaired development of the ventricular wall and septum.^93^, ^231^, ^232^ This was initially unexpected and showed the importance of calreticulin in cardiac function and development. Two decades of studying calreticulin in the heart very well illustrate and exemplify the concept of co‐dependence between calreticulin and Ca^2+^.

### 7.1 Loss of calreticulin Ca^*2*+^ pool in the heart

Cardiac development is a well‐controlled molecular and morphogenetic event where Ca^2+^ plays an important signalling role that affects many transcriptional processes during cardiogenesis.^233^, ^234^ Any perturbations of Ca^2+^‐dependent signalling pathways can have devastating consequences in the form of congenital heart disease.^233^

The whole body inactivation of calreticulin gene in mice is embryonic lethal at E18.5.^93^, ^231^ In vitro and in vivo biochemical, cell biological and animal studies of calreticulin indicate that Ca^2+^ handling by calreticulin is the key cause of embryonic lethality of calreticulin‐deficient mice.^93^, ^235^ Since Ca^2+^‐dependent transcriptional mechanisms play crucial roles during cardiac development and pathology,^233^, ^234^, ^236^, ^237^ we tested whether loss of the calreticulin Ca^2+^ pool in cardiomyocytes is responsible for embryonic lethality in the absence of calreticulin. This idea was validated by the creation of a ‘rescue’ mouse model with cardiac‐specific expression of a constitutively active form of calcineurin.^235^, ^238^ Calcineurin is a strictly Ca^2+^‐dependent serine/threonine phosphatase that regulates the activity of transcription factor NF‐AT. Increased expression of calcineurin in cardiomyocytes results in cardiac hypertrophy.^239^ Sustained Ca^2+^ release from the ER is required to activate calcineurin phosphatase activity,^240^ but deletion of the C‐terminal domain of calcineurin produces a constitutively active phosphatase even in the absence of Ca^2+^ release from the ER.^241^ Expression of constitutively active calcineurin in calreticulin‐deficient cardiomyocytes rescued *Calr* ^*−/−*^ mice from embryonic lethality^235^ indicating an essential role for the calreticulin Ca^2+^ pool in Ca^2+^‐dependent transcriptional events during cardiac development.^232^, ^235^, ^238^, ^242^, ^243^ This remarkable finding illustrates a key role of calreticulin in managing the Ca^2+^ pool required in regulating Ca^2+^‐dependent transcriptional pathways. Additional studies demonstrated that calreticulin similarly plays a role in the transcription of genes regulated by glucocorticoid receptor and other nuclear hormone receptors,^244^, ^245^, ^246^, ^247^, ^248^ extending calreticulin influence beyond the ER and impacting on many cellular functions.

CALRdel52, the calreticulin gene variant associated with MPN has all the negative charges of the calreticulin Ca^2+^ binding C‐domain converted to positive charges thus influencing the ER calreticulin Ca^2+^ pool.^94^, ^95^ Not surprisingly, replacement of the wild‐type calreticulin gene by knock‐in of the *CALR*del52 variant in all cells in mice (loss of calreticulin Ca^2+^ pool) results in embryonic lethality^110^ thus validating the original finding that the loss of calreticulin Ca^2+^ pool in cardiomyocytes is responsible for embryonic lethality.^93^, ^235^ Taken together, these findings firmly establish that calreticulin Ca^2+^ pool is well integrated into Ca^2+^‐dependent transcriptional events. Importantly, the loss of high‐capacity Ca^2+^ binding to calreticulin and consequently the loss of the calreticulin ER Ca^2+^ pool makes the protein functionally ineffective and leads to pathology.

### 7.2 Increased calreticulin Ca^*2*+^ pool in the heart

Although the abundance of calreticulin and the size of the calreticulin Ca^2+^ pool is initially is highly in the embryonic heart, the abundance of calreticulin and concomitantly the size of the calreticulin Ca^2+^ pool, sharply decreases in postnatal and adult cardiomyocytes.^93^ This turns out to be important for the development of cardiac conductive system because maintaining a high level of calreticulin Ca^2+^ pool in the postnatal heart results in impaired systolic function, sinus bradycardia and prolonged atrioventricular (AV) node dysfunction with progressive prolongation of the P‐R interval that result in complete heart block and sudden death in mice.^249^ This is reminiscent of a complete heart block seen in children.^250^ Furthermore, in the embryonic stem (ES) cell model of cardiogenesis the increasing calreticulin Ca^2+^ pool or knocking‐down the InsP_3_R/Ca^2+^ channel (Ca^2+^ export protein) prevents proper development of ES‐derived pacemaker cells.^236^ Thus, the role of calreticulin in delivering Ca^2+^ to the InsP_3_R is essential for proper development of pacemaker activity during early cardiogenesis and foetal life.

The increased of the calreticulin Ca^2+^ pool in the adult heart enhances mechanical work potential of cardiomyocytes and activates the IRE1α branch of the UPR all leading to cardiac fibrosis and heart failure.^251^, ^252^ Increased mechanical work of cardiomyocytes triggers activation of UPR in cardiac fibroblasts leading to fibrosis and cardiac remodelling.^252^ Remarkably, blocking the activation of the UPR pathway by tauroursodeoxycholic acid (TUDCA) prevents cardiac fibrosis.^246^ The mechanisms of action of TUDCA on UPR signalling are not well understood but likely involve TUDCA‐dependent alteration of cellular and/or ER Ca^2+^ (managed by calreticulin).^253^, ^254^, ^255^

### 7.3 Calreticulin Ca^*2*+^ pool versus calsequestrin Ca^*2*+^ pool

Cardiac and skeletal muscle are both highly specialized tissues sustaining mechanical function of muscle. Muscle contraction is triggered by Ca^2+^ release from the junctional SR. The SR membrane contains a number of highly specialized Ca^2+^ handling proteins, including calsequestrin, supporting exclusively muscle excitation–contraction coupling. Muscle cells also contain functional ER (calreticulin domicile) associated with house‐keeping jobs such as Ca^2+^ and transcriptional signalling, protein quality control, lipid metabolism and ER stress responses, to name a few.^2^ Thus, it is entirely predictable that loss of the ER‐associated calreticulin Ca^2+^ pool in cardiomyocytes explains impairment of heart development and embryonic lethality.^93^, ^231^ In contrast mice lacking cardiac calsequestrin (loss of the SR calsequestrin‐associated Ca^2+^ pool) are viable and fertile but only manifest dysfunction in RyR2 channel and conduction abnormalities.^256^, ^257^ This further illustrates that cardiomyocytes not only have two functionally distinct membrane systems (ER and SR)^2^ but most importantly they have two functionally different Ca^2+^ pools; one highly specialized calsequestrin Ca^2+^ pool (driving excitation–contraction coupling) and an ER calreticulin Ca^2+^ pool that is responsible for handling many Ca^2+^‐dependent cellular processes (transcription, stress responses, protein quality control and turnover, lipid metabolism).

In summary, one lesson from the heart is that cardiac cells are unique because there is functional compartmentalization of Ca^2+^ in cardiomyocytes with the SR Ca^2+^ being specialized for muscle mechanical function and the ER Ca^2+^, handled by calreticulin for general cellular functions. Calsequestrin Ca^2+^ pool evolved as Ca^2+^ pool dedicated to supply Ca^2+^ for excitation–contraction coupling. Another lesson from the heart is that the Ca^2+^ pool under the control of calreticulin is not only essential for cardiogenesis and development of muscle conductive system, but most importantly, it impacts on cells Ca^2+^ and transcriptional signalling, protein quality control, energy metabolism and ER stress responses. The calreticulin Ca^2+^ pool must be tightly controlled as any increase or decrease in calreticulin Ca^2+^ pool results in pathologies.

## 8 CONCLUDING THOUGHTS

**FIGURE 8:**
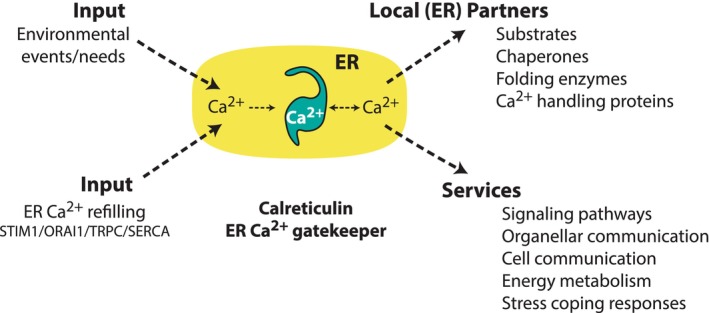
Calreticulin as the ER Ca^2+^ gatekeeper. As the Ca^2+^ gatekeeper calreticulin maintains ER Ca^2+^ supply, manages access to Ca^2+^ and how Ca^2+^ is used. Ca^2+^ is supplied to the ER from the extracellular space and cytoplasm by combined action of SOCE and SERCA. Depending on the environmental conditions/cellular needs Ca^2+^ is released from the Ca^2+^ gatekeeper calreticulin to manage ER ‘local’ events or other needed ‘services’ including stress responses, energy metabolism and cellular and organellar communication.

Ca^2+^ has emerged through evolution as a ubiquitous signalling molecule and integral part of the physiology and biology of the organism. Eukaryotic cells developed Ca^2+^ stores system of the ER, which serves as an internally controlled source of Ca^2+^ for organellar and cellular communications, essential for survival. The story of calreticulin begun over 40 years ago with a question: how is Ca^2+^ handled in the lumen of the ER? This led to the discovery of calreticulin, a protein that today is universally accepted as the major Ca^2+^ handling protein in all cells that have ER. Over three decades of studying calreticulin demonstrates that calreticulin plays the role of the ER Ca^2+^ gatekeeper (Figure 8). Both the role of calreticulin as the ER Ca^2+^ gatekeeper and the interdependence of calreticulin and Ca^2+^ form the foundation of ER Ca^2+^ signalling and, therefore, influences many cellular processes (Figure 8). The protein maintains ER Ca^2+^ supply under different physiological conditions, manages access to Ca^2+^, how Ca^2+^ is used depending on the environmental events, and makes sure Ca^2+^ is not misused. The ER is an extensive network of membranes that occupies a major proportion of the CRN, and calreticulin, as the ER Ca^2+^ gatekeeper, partitions Ca^2+^ to different regions of the CRN for many cellular functions and needs. This explains why calreticulin turns up in numerous studies involving cell function.

## AUTHOR CONTRIBUTIONS

**Marek Michalak:** Conceptualization (lead); data curation (lead); formal analysis (lead); funding acquisition (lead); investigation (lead); project administration (lead); visualization (lead); writing – original draft (lead); writing – review and editing (lead).

## FUNDING INFORMATION

Research in our laboratory is supported by a generous donation from the Kenneth and Sheelagh McCourt family; University Hospital Foundation; Canadian Institutes of Health Research; Natural Sciences & Engineering Research Council of Canada; Canada Foundation for Innovation; Multiple Sclerosis Society; Women and Children's Health Research Institute; SynAD.

## CONFLICT OF INTEREST STATEMENT

The author declares no conflict of interest.

## Data Availability

Not applicable
